# Inhibitor of DNA Binding Protein 2 (ID2) Mediates the Anti-Proliferative and Pro-Differentiation Effects of Insulin-like Growth Factor-1 (IGF-1)

**DOI:** 10.3390/life14121663

**Published:** 2024-12-16

**Authors:** Rebecca Ssengonzi, Yuye Wang, Jiayi Zhou, Yukako Kayashima, W. H. Davin Townley-Tilson, Balaji Rao, Qing Ma, Nobuyo Maeda-Smithies, Feng Li

**Affiliations:** 1 Department of Pathology and Laboratory Medicine, The University of North Carolina at Chapel Hill, Chapel Hill, NC 27599, USA; rssengo@email.unc.edu (R.S.); yuye@email.unc.edu (Y.W.); yukaya@email.unc.edu (Y.K.); davin_townley_tilson@med.unc.edu (W.H.D.T.-T.); maqing@email.unc.edu (Q.M.); nobuyo@med.unc.edu (N.M.-S.); 2 Department of Nutrition, Gillings School of Global Public Health, University of North Carolina at Chapel Hill, Chapel Hill, NC 27599, USA; jz0105@email.unc.edu; 3 Department of Chemical and Biomolecular Engineering, Golden LEAF Biomanufacturing Training and Education Center, North Carolina State University, Raleigh, NC 27606, USA; bmrao@ncsu.edu

**Keywords:** ID2, IGF-1, HTR8/SVneo cell, placenta, preeclampsia

## Abstract

In preeclampsia (PE), impaired trophoblast proliferation and differentiation are thought to cause abnormal placentation and subsequent clinical manifestations of the disease, i.e., hypertension, proteinuria, and end-organ damage. Insulin-like growth factor-1 (IGF-1) influences trophoblast cell function; however, the mechanism of IGF-1’s action on trophoblasts is not understood well. Inhibitor of DNA binding protein 2 (ID2) is involved in trophoblast differentiation and implicated in many processes disrupted in PE, including placental development, vascular differentiation, and angiogenesis. We hypothesized that IGF-1 regulates trophoblast proliferation and differentiation via ID2. Immortalized human first trimester trophoblast cells (HTR-8/SVneo) were treated with IGF-1 for 24 h after serum starvation. ID2 mRNA and protein were measured, as well as trophoblast cell viability, proliferation, tube formation, and migration. IGF-1 decreased ID2 expression in a dose-dependent manner. IGF-1 decreased trophoblast proliferation but increased cell viability, differentiation, and migration. ID2 overexpression mitigated the effects of IGF-1 on trophoblast cells. These data suggest that IGF-1 could regulate trophoblast proliferation and differentiation through ID2. The dysregulation of ID2-mediated IGF-1 signaling in trophoblast cells could be involved in the pathogenesis of pregnancy disorders like uterine growth restriction and PE.

## 1. Introduction

The appropriate proliferation and differentiation of trophoblast cells are pivotal for implantation and subsequent placentation in healthy pregnancy [1]. Impaired trophoblast functions are involved in many common complications of pregnancy, including preeclampsia (PE) and uterine growth restriction [2]. Various molecules have been shown to influence trophoblast biology, and accumulating evidence suggests that inhibitor of DNA binding protein 2 (ID2)—a marker of stemness—is involved in trophoblast stem cell proliferation and differentiation [3]. ID2 is a member of the inhibitor of differentiation (ID) protein family, which is characterized by its similar structure to the basic helix-loop-helix (bHLH) family of transcription factors [4]. Unlike the bHLH family, ID proteins like ID2 lack a basic DNA-binding domain, but they can still heterodimerize with bHLH transcription factors [4]. ID2 heterodimerization with bHLH inhibits differentiation, as bHLH can no longer bind DNA to drive the transcription of gene targets [5]. A sustained high expression of ID2 inhibits trophoblast cell differentiation. Gultice et al. reported that the rat trophoblast cell line Rcho-1, which represents an isolated trophoblast population committed to the giant cell lineage (equivalent to human extravillous trophoblast cells), had impaired differentiation under hypoxic conditions [6]. This impaired differentiation was attributed to hypoxia inhibiting the downregulation of ID2 [6]. In addition, Selesniemi et al. reported that, in the mouse labyrinthine placental progenitor cell line SM10, ID2 overexpression prevents differentiation, while ID2 knockdown promotes differentiation [3].

Insulin-like growth factor-1 (IGF-1)—a single-chain basic polypeptide growth factor containing 70 amino acid residues—plays an important role in regulating cell proliferation, differentiation, and survival [7,8]. IGF-1 is also involved in pregnancy complications, including uterine growth restriction, gestational diabetes mellitus, and PE, but the precise role of IGF-1 in pregnancy is not clear [9,10,11]. Recently, Lai et al. showed that IGF-1 enhanced the proliferation and invasion of trophoblast cells, and that silencing IGF-1 reduced these effects [12]. Furthermore, placental-specific knockdown of IGF-1 by lentivirus caused PE-like phenotypes in mice [12]. Their results suggest that IGF-1 is important for trophoblast function, and that its deficiency could result in PE or other pregnancy complications. The effect of IGF-1 on ID2 in trophoblast cells is not clear, though it has been tested before in other cell types; in 32D murine hemopoietic cells, IGF-1 signaling increased ID2, which correlated with inhibited cell differentiation [13]. HTR-8/SVneo (HTR8) is a well-established human first-trimester extravillous trophoblast cell line that displays progenitor cell characteristics, i.e., self-renewal and ID2 expression [14,15,16]. In this study, we tested whether IGF-1 executes its function on trophoblast cells through ID2.

## 2. Methods

Cell culture: The HTR8 trophoblast cell line was kindly provided by Dr. C.H. Graham, Queen’s University, Kingston, Ontario, Canada [14], and maintained inRPMI-1640 medium supplemented with 5% fetal bovine serum (FBS) [17]. The cells were starved for 24 h in 0% FBS media, then treated with IGF-1 (GF306, EMD Millipore Corp., Burlington, MA, USA) in 0% FBS media at serial doses spanning from physiological (10 ng/mL) to supraphysiological (100 and 1000 ng/mL) levels for 24 h [18,19]. At the end of the treatment, cells were collected for western blot and quantitative RT-PCR (qRT-PCR) analyses. Different batches of HTR8 cells were treated with 0 ng/mL IGF-1, 100 ng/mL IGF-1, 20 µM U0126 (AAJ61246MB, ThermoFisher Scientific, Waltham, MA, USA), 10 µM LY294002 (PHZ1144, ThermoFisher Scientific, Waltham, MA, USA), or the combination of IGF-1 and U0126 or LY294002, respectively. After 24 h of treatment, cells were collected for qRT-PCR analysis.

Cell viability assay: HTR8 cells (10^4^ per well) were seeded in each well of a 96-well plate (35307, Falcon, Corning, NY, USA) in 100 µL of 5% FBS media and allowed to attach overnight. Once cells had reached greater than 90% confluence, they were starved with 0% FBS for 24 h. The media in each well were discarded, then replaced with 100 µL of 0% FBS media with the different concentrations of IGF-1. After 24 h of treatment, CCK-8 solution (96992, Sigma-Aldrich, St. Louis, MO, USA) was added to each well (final 1/10 dilution) and incubated for 1 h, as described previously [20,21]. The absorbance was measured at 450 nm using a BioTek Synergy HT microplate reader (BioTeK, Agilent, Santa Clara, CA, USA).

Transfection: HTR8 cells (2 × 10^4^) were seeded in 24-well plates (353047, Falcon, Corning, NY, USA) with 0.5 mL of 5% FBS media and allowed to attach overnight. Each well was transfected with 0.125 µg of either control (PS100092, OriGene Technologies, Inc., Rockville, MD, USA) or ID2 (SC118791, OriGene Technologies, Inc., Rockville, MD, USA) plasmids and lipofectamine LTX with Plus reagents (A12621, ThermoFisher Scientific, Waltham, MA, USA) following the manufacturers’ instructions. After 24 h of transfection, the media were changed back to growing media (5% FBS) for further experiments.

Cell proliferation assay: Endogenous ID2 expression: HTR8 cells (10^4^ per well) were seeded in each well of a 96-well plate (35307, Falcon, Corning, NY, USA) with 100 µL of 5% FBS media and allowed to attach overnight. Once cells had reached greater than 90% confluence, they were starved with 0% FBS for 24 h. The media in each well were discarded, then replaced with 100 µL of 0% FBS media with the different concentrations of IGF-1. After 24 h of treatment, the Click-iT 5-ethynyl-2′deoxyuridine (EdU) proliferation assay for microplates (C10499, ThermoFisher Scientific, Waltham, MA, USA) was conducted following the manufacturer’s instructions, and fluorescence was measured using a BioTek Synergy HT microplate reader. Transfection: after 48 h of transfection, cells from each plasmid group were trypsinized and replated on a 96-well plate with 5µM EdU (C10499, ThermoFisher Scientific, Waltham, MA, USA), and the three different concentrations of IGF-1 were placed in 0% FBS media. After 72 h of transfection, the Click-iT EdU proliferation assay for microplates (C10499, ThermoFisher Scientific, Waltham, MA, USA) was conducted following the manufacturer’s instructions, and fluorescence was measured using a BioTek Synergy HT microplate reader.

Tube formation assay: Endogenous ID2 expression: HTR8 cells were grown in a 24-well plate (353047, Falcon, Corning, NY, USA) with 5% FBS media until they were greater than 90% confluent. Then, they were serum starved in 0% FBS media for 24 h. Afterwards, the cells were treated with the three different concentrations of IGF-1 for 24 h. Tube formation was visualized with a Nikon TMS inverted microscope, and images were captured using a Nikon Digital Sight 1000 camera. ImageJ was used to measure tube diameter. Transfection: after 48 h of transfection, cells from each plasmid group were treated with the different concentrations of IGF-1 in 0% FBS media. After 72 h of transfection, tube formation was visualized with a Nikon TMS inverted microscope and images were captured using Nikon Digital Sight 1000 camera. ImageJ was used to measure tube diameter.

Wound healing assay: Endogenous ID2 expression: HTR8 cells were grown in a 24-well plate (353047, Falcon, Corning, NY, USA) with 5% FBS media until they were greater than 90% confluent. Then, they were serum starved in 0% FBS media for 24 h. Afterwards, cells were scratched along the midline of the well with a 200 µL pipette tip (4845, Universal Fit Pipet Tips, Corning, NY, USA, approximately 900 µm in diameter) and rinsed with PBS [21]. Scratched wells were treated with the three different concentrations of IGF-1 in 0% FBS media for 24 h. Cell migration into the wound was visualized with a Nikon TMS inverted microscope, and images were captured using a Nikon Digital Sight 1000 camera. Transfection: 2 × 10^4^ HTR8 cells were seeded in 24-well plates (353047, Falcon, Corning, NY, USA) in 0.5 mL of 5% FBS media and allowed to attach overnight. Each well was transfected with 0.5 µg of either control or ID2 plasmids. After 48 h of transfection, cells were scratched along the midline of the well with a 200 µL micropipette tip and rinsed with PBS [21]. The scratched wells were treated with the three different concentrations of IGF-1 in 0% FBS media. After 72 h of transfection, cell migration into the wound was visualized with a Nikon TMS inverted microscope, and images were captured using a Nikon Digital Sight 1000 camera.

Mice: Female/male wild type (WT) C57BL/6J mice were housed in standard cages on a 12 h light/dark cycle and were allowed free access to food and water. All experiments were carried out in accordance with the National Institute of Health’s guidelines for the use and care of experimental animals, as approved by the IACUC of the University of North Carolina at Chapel Hill.

Two females were housed with one male mouse together around 4–5:00 pm, and the virginal plugs were checked at the next morning around 8:00 am. The day vaginal plugs were detected was defined as 0.5 days post-coitus (dpc), and the female mice were randomly enrolled into either control (vehicle, PBS) or IGF-1 treatment groups. IGF-1-treated mice were administered IGF-1 at a dose of 20 µg/kg/day via intraperitoneal injection (the volume injected was 100 µL) [22].

A stock solution was prepared by dissolving 50 µg of IGF-1 (R&D system, 791-MG-050) in 1 mL PBS (pH = 7.4). Immediately before injection, the stock solution was diluted to make the working solution.

At 12.5 dpc (when the placenta is fully formed), uterine tissues were collected, and placentas were carefully isolated and were either snap frozen or fixed in 4% paraformaldehyde. Snap frozen placentas were subjected to a qRT-PCR assay, while fixed tissues were tested for immunofluorescence.

Immunofluorescence: The placenta samples were paraffin-embedded, cut into 5-μm sections, and mounted on slides. After deparaffinization, placenta tissue sections were treated with 10 mM citrate buffer (pH 6.0) for antigen retrieval. After blocking with 10% normal chicken serum and 0.1% BSA at room temperature for 1 h, the sections were incubated with rabbit monoclonal anti-Keratin 17 (1:200; Cat#12509, Cell Signaling Technology Inc., Danvers, MA, USA) and mouse monoclonal anti-ID2 (1:200; Invitrogen, Cat# MA5-32891, Rockford, IL, USA) at 4 °C overnight. After 3 washes with PBS, the sections were then incubated with Alexa Fluor 488-conjugated goat anti-rabbit IgG (1:500; Invitrogen, Carlsbad, CA, USA) or Alexa Fluor 596-conjugated goat anti-mouse IgG (1:500; Invitrogen, Carlsbad, CA, USA) at room temperature for 2 h. After washing with PBS, the slides were prepared and mounted using Fluoromount-G, 4′,6-diamidino-2-phenylindole (DAPI) (Southern Biotech, Cat. # 0100-20, Birmingham, AL, USA) to detect nuclei. Images were captured on an Olympus fluorescent microscope (Evident Scientific, Inc., Waltham, MA, USA) using a 10×/0.4 or 20× or 40×PH objective at 1.0-fold magnification.

Western Blot: A lysis buffer—0.1% Triton X-100 (X198-07, Avantor Performance Materials, Center Valley, PA, USA) in PBS—was added to the cells for 30 min. The cells were scraped from culture plates and centrifuged to separate debris. The protein concentrations in cell lysates were determined by a Pierce BCA Protein Assay kit (23225, ThermoFisher Scientific, Waltham, MA, USA). Total protein levels from 20 to 60 µg/lane were subjected to 4–20% SDS-PAGE, then electrotransferred onto PVDF membranes. The chemiluminescent intensities of the targeted protein bands were captured using the ODYSSEY^®^ FC system and evaluated using Image Studio Software 2.0 (LI-COR Biosciences, Lincoln, NE, USA). The individual protein level of each sample was quantified by normalizing its intensity to the β-actin in the same sample and expressed relative to the levels of the respective control group, the mean of which was set as 1. The antibodies used in the study included ID2 (MA5-32891, Invitrogen, Waltham, MA, USA) and β-actin (5125, Cell Signaling Technology, Danvers, MA, USA).

qRT-PCR: The total RNA from the cells was extracted using TRIzol Reagent (15596018, Invitrogen, Waltham, MA, USA) following the manufacturer’s instructions. A BioTek Synergy HT microplate reader was used to determine RNA concentration. mRNA was quantified with TaqMan real-time qRT-PCR (7500 real-time PCR system, Applied Bio-systems, Foster City, CA, USA) by using a one-step RT-PCR Kit (Bio Rad, Hercules, CA, USA) with GAPDH (human cells) and Actb (mouse tissues) as reference genes in each reaction. The 2^−ΔΔCt^ method was used for comparing the data [17,23]. Primer and probe sequences are listed in Table 1.

Statistical analysis: The data are presented as mean ± standard error of the mean unless otherwise stated. A multifactorial analysis of variance test was used with the program JMP 17.0 (SAS Institute Inc., Cary, NC, USA). Post hoc analyses were performed using the Tukey–Kramer Honest Significant Difference test.

## 3. Results

### 3.1. IGF-1 Decreases ID2 Expression in Trophoblasts in a Dose-Dependent Manner

**Figure 1:**
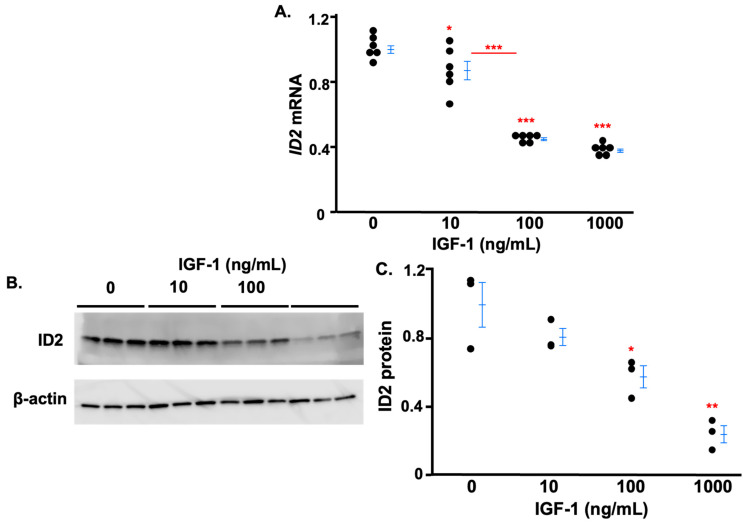
IGF-1 decreases ID2 expression in trophoblasts in a dose-dependent manner. ID2 mRNA in HTR8 cells after 24 h of IGF-1 treatment (**A**). Western blot (**B**) and densitometric quantitation (**C**) of ID2 proteins in HTR8 cells after 24 h of IGF-1 treatment. Asterisks indicate groups that are significantly different from 0 ng/mL IGF-1 or significantly different from each other (compared with red line): * *p* < 0.05, ** *p* < 0.01, *** *p* < 0.0001. (**A**) (n = 6), (**B**) (n = 3), and (**C**) show representative experiments which were repeated twice. Data are presented as mean ± standard deviation in (**C**).

After 24 h, the IGF-1 treatments at doses of 10, 100, and 1000 ng/mL decreased mRNA levels of ID2 by approximately 13%, 56%, and 63%, respectively, while there was no significant difference between the cells treated with IGF-1 at a dose of 100 ng/mL or 1000 ng/mL (Figure 1A). Consistent with mRNA data, increasing IGF-1 decreased protein levels of ID2 in a dose-dependent manner in HTR8 cells (Figure 1B,C). Neither the inhibition of MEK1/2 (U0126) nor the inhibition of PI3K (LY-294002) at the dose we applied abolished the effects of IGF-1 on the expression of ID2 (Supplementary Figure S2).

### 3.2. IGF-1 Inhibits the Proliferation of Trophoblasts Which Is Rescued by ID2 Overexpression

**Figure 2:**
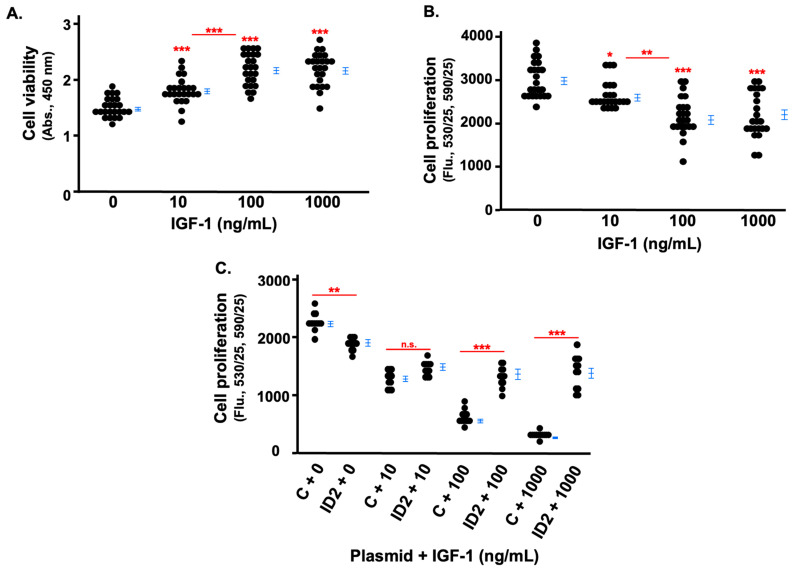
IGF-1 inhibits the proliferation of trophoblasts which is rescued by ID2 overexpression. HTR8 cell viability (**A**) and proliferation (**B**) after 24 h of IGF-1 treatment. Cell viability was measured by absorbance (450 nm), and cell proliferation was measured by fluorescence (530/25, 590/35). The proliferation of transfected (control, noted as “C” in the horizontal axis, or ID2 plasmids) HTR8 cells after 24 h of IGF-1 treatment (**C**). Asterisks indicate groups that are significantly different from 0 ng/mL IGF-1 treatment or groups significantly different from each other (compared with red line): * *p* < 0.05, ** *p* < 0.01, *** *p* < 0.0001, n.s. is not significant. (**A**) (n = 24), (**B**) (n = 24), and (**C**) (n = 12) show representative experiments which were repeated twice.

We first tested the effects of IGF-1 on cell viability and found that IGF-1 did not have a cytotoxic effect on HTR8 cells at any of the three doses; cell viability was not decreased, but was instead increased by IGF-1 treatment for 24 h (Figure 2A). Afterwards, we tested the direct effect of IGF-1 on the proliferation of HTR8 cells using a Click-iT EdU proliferation assay, which detects newly synthesized DNA. We found that increasing IGF-1 inhibited the proliferation of HTR8 cells in a dose-dependent manner. After 24 h, the proliferation of HTR8 cells treated with 10, 100, and 1000 ng/mL of IGF-1 was approximately 87%, 74%, and 70% of non-treated cells, respectively (Figure 2B).

Then, we investigated the role of ID2 in the anti-proliferative effects of IGF-1 on HTR8 cells. First, we transfected cells with control and ID2 plasmids, and the ID2 plasmids increased ID2 protein levels more than 20 times compared to the control plasmids (Supplementary Figure S1). The proliferation of IGF-1-treated HTR8 cells transfected with ID2 plasmids significantly increased at 100 and 1000 ng/mL compared to IGF-1-treated HTR8 cells transfected with control plasmids (Figure 2C). The significant difference in proliferation between HTR8 cells transfected with control and ID2 plasmids in the absence of IGF-1 could be attributed to off-target effects of the plasmids (Figure 2C).

### 3.3. IGF-1 Promotes Tube Formation in Trophoblasts Which Is Mitigated by ID2 Overexpression

**Figure 3:**
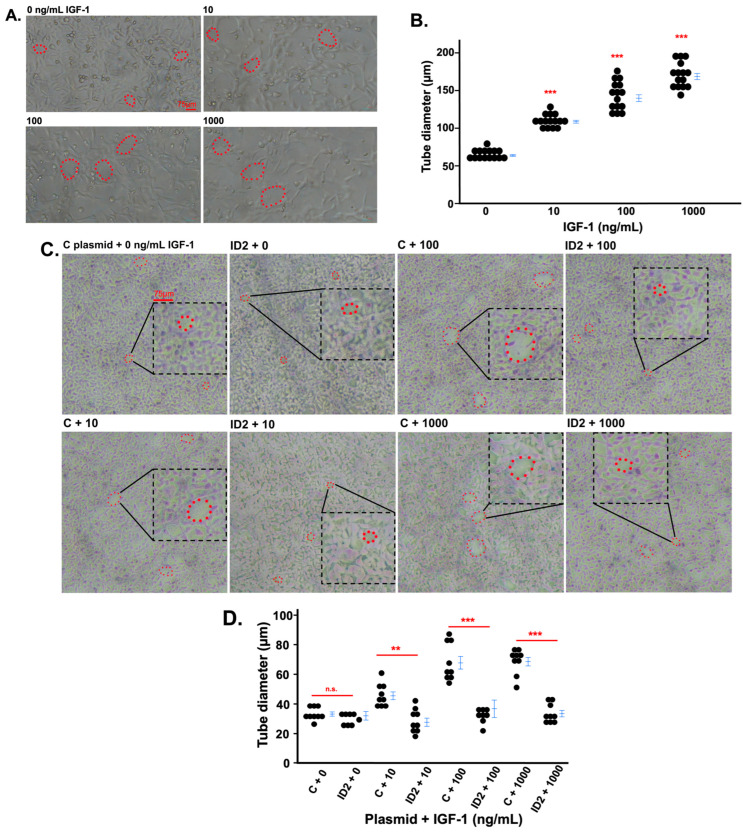
IGF-1 promotes tube formation in trophoblasts which is mitigated by ID2 overexpression. Light microscopy (4×) images showing tube formation in HTR8 cells after 24 h of IGF-1 treatment (**A**). (**B**) is the quantification of tube diameters in (**A**). Red dots outline representative tubes, i.e., the way the cells form capillary-like structures with neighboring cells. Light microscopy images showing tube formation in transfected (control, noted as “C” in the figures, or ID2 plasmids) HTR8 cells after 24 h of IGF-1 treatment (**C**). (**D**) is the quantification of tube diameters in (**C**). Asterisks indicate groups that are significantly different from 0 ng/mL IGF-1 or groups significantly different from each other (compared with the red line). ** *p* < 0.01, *** *p* < 0.0001, n.s. is not significant. (**A**) shows one representative image for each treatment group. Three tube diameters were quantified per image (n = 5) (**B**). (**C**) shows one representative image for each treatment group. Three tube diameters were quantified per image (n = 3) (**D**). All experiments were repeated twice.

We determined the effects of IGF-1 on the tube formation of HTR8 cells. The three doses of IGF-1 increased the size of the tubes (Figure 3A,B). However, the tube formation of IGF-1-treated HTR8 cells transfected with ID2 plasmid significantly decreased at 100 and 1000 ng/mL compared to IGF-1-treated HTR8 cells transfected with control plasmids (Figure 3C,D).

### 3.4. IGF-1 Promotes Trophoblast Migration Which Is Mitigated by ID2 Overexpression

**Figure 4:**
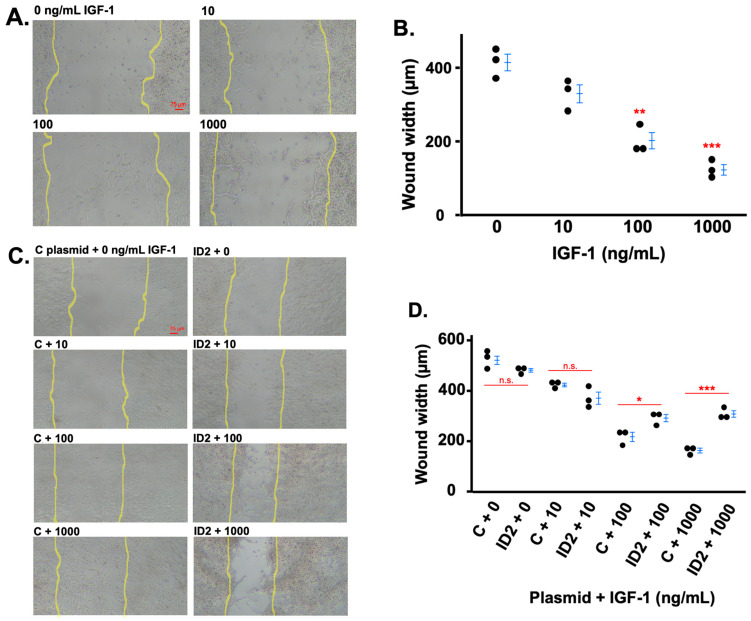
IGF-1 promotes trophoblast migration which is mitigated by ID2 overexpression. Light microscopy (4×) images showing cell migration after 24 h of IGF-1 treatment (**A**). The yellow lines approximate where the original injury was (based on dense cell growth as cells were >90% confluent before scratching). (**B**): The quantification of wound width in (**A**). Light microscopy showing cell migration in transfected (control, noted as “C” in the figures, or ID2 plasmids) HTR8 cells after 24 h of IGF-1 treatment (**C**). (**D**): The quantification of wound width in (**C**). Asterisks indicate groups that are significantly different from 0 ng/mL IGF-1 treatment or groups significantly different from each other (compared with red line): * *p* < 0.05, ** *p* < 0.01, *** *p* < 0.0001, n.s. is not significant. (**A**,**B**) show one representative image (n = 3) for each treatment group. All experiments were repeated twice. Data are presented as mean ± standard deviation in (**B**,**D**).

We determined the effects of IGF-1 on the migration of HTR8 cells utilizing the wound healing method. The three doses of IGF-1 markedly decreased the width of the wounded area to approximately 20%, 50%, and 70% of non-treated cells, respectively (Figure 4A,B). We investigated the role of ID2 in the IGF-1-induced cell migration of HTR8 cells. In the absence of IGF-1, there was no significant difference in the wound width of HTR8 cells transfected with ID2-overexpressing plasmids compared to HTR8 cells transfected with control plasmids (Figure 4C,D). However, the wound width of IGF-1-treated HTR8 cells transfected with ID2 plasmids was significantly larger at 100 and 1000 ng/mL compared to IGF-1-treated HTR8 cells transfected with control plasmids (Figure 4C,D).

### 3.5. IGF-1 Administration to WT Female Mice Decreases Id2 in Placentas

**Figure 5:**
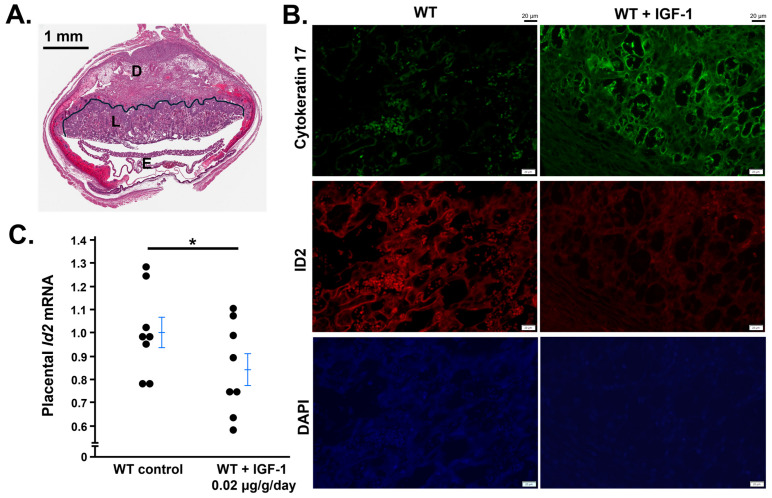
IGF-1 treatment decreases ID2 in WT mouse placentas. (**A**) Structure of the placenta at 12.5 days post coitus, D: decidua, L: labyrinthine where the images for (**B**). were taken, E: embryo. Light microscopy, 4×. (**B**) IGF-1 treatment for 12 days decreased placental immunostaining of ID2 and increased immunostaining of Cytokeratin 17. Fluoromount-G, 4′,6-diamidino-2-phenylindole (DAPI) is used to detect nuclei. Wide-field microscopy, 20×. (**C**) mRNA levels of ID2 in the placentas from WT dams with or without IGF-1 treatment. One point represents a placenta randomly collected from a pregnant mouse. * *p* < 0.05.

IGF-1 treatment starting at the beginning of pregnancy for 12 days decreased immunostaining of ID2 but increased the immunostaining of Cytokeratin 17 (Figure 5B), which is a marker for trophoblast cell differentiation [24], and this treatment regimen decreased mRNA levels of ID2 in placentas determined by qRT-PCR (Figure 5C).

## 4. Discussion

While IGF-1 has previously been found to influence proliferation and differentiation in various cell types, including trophoblast cells, no study has investigated ID2 as a potential mediator of IGF-1 in these cellular processes [12,25,26,27,28,29,30]. In this study, we have demonstrated that IGF-1 downregulation of ID2—at both the mRNA and protein levels—could mitigate the proliferation of HTR8 cells while promoting their differentiation. In addition, ID2 overexpression mitigates the anti-proliferative, pro-differentiation effects of IGF-1 on HTR8 cells. Furthermore, our in vivo data demonstrates that IGF-1 treatment decreased ID2 and increased Keratin 17. These data suggest that ID2 at least partially mediates the effects of IGF-1 on HTR8 cell proliferation and differentiation.

We demonstrated that all three doses of IGF-1 treatment affected HTR8 cells. While it is not clear if IGF-1 still executes the function at lower doses (<10 ng/mL), it is worthwhile to investigate the effects of lower doses of IGF-1.

IGF-1 is expressed by almost all cells, and the main source of circulating IGF-1 is from hepatocytes, which are regulated by growth hormones from the pituitary gland [31]. The bioactivity of IGF-1 is modulated by IGF-binding proteins (IGFBPs), and IGF-1 executes its cellular function through IGF-1 receptors (IGF1-R) [32]. IGF-1/IGF1-R signaling plays an important role in the female reproductive system. For example, IGF-1 deficiency leads to impaired granulosa cell proliferation [33]. Several lines of evidence suggest that IGF-1 is involved in pregnancy complications, including PE. One study reported that IGF-1 was lower and IGFBP-1 was higher in PE patients compared to normotensive pregnant women, and these changes were correlated with the severity of PE [34]. However, the authors did not clarify during which stage of gestation IGF-1 and IGFBP-1 were measured. Later, Ning et al. demonstrated that preeclamptic women had lower plasma IGF-1 and IGFBP-1 compared to normotensive women at 13 weeks of gestation [35]. Furthermore, in experimental animal studies, low IGF-1 caused pregnancy problems: Lorenzini et al. observed that IGF-1-deficient female mice had decreased litter sizes compared to wild-type females; however, it is not clear if these dams had hypertension and proteinuria [36]. Lai et al. reported that female mice with reduced IGF-1 expression established by a lentiviral-mediated, placental-specific knockdown developed PE-like phenotypes during pregnancy, including elevated blood pressure, proteinuria, and decreased fetal weight [12]. The authors also found that IGF-1 enhanced the proliferation, invasion, and angiogenesis of trophoblast cells in vitro [12]. Taken together, lower-than-normal levels of IGF-1 could negatively affect trophoblast biology, which may contribute to PE pathogenesis. In contrast, Irani et al. reported that women undergoing euploid blastocyst transfer with elevated serum IGF-1 concentrations may be at increased risk of pregnancy loss [37]. During early placentation, trophoblast progenitors must sufficiently proliferate before they start to differentiate. If trophoblast progenitors are exposed to abnormally high levels of IGF-1, these cells could have impaired proliferation and immature differentiation, which could lead to defective placentation and adverse pregnancy outcomes. In contrast, low IGF-1 levels during the trophoblast cell differentiation stage could impair trophoblast cell differentiation, causing insufficient placentation and subsequent pregnancy complications. Overall, the precise mechanism by which IGF-1 affects pregnancy is not clear, as both high and low IGF-1 levels could have detrimental effects.

Here, we tested the effects of physiological and supraphysiological levels of IGF-1 on ID2 because of the pivotal role of ID2 in trophoblast cell biology. We found that IGF-1 impeded HTR8 cell proliferation at all doses we applied, and overexpressing ID2 mitigated the inhibition effect of IGF-1. Lai et al. found that HTR8 cell proliferation was decreased after silencing IGF-1 and concluded that IGF-1 promoted HTR8 cell proliferation [12]. The method they utilized to determine cell proliferation was a CCK-8 kit, which detects the number of live cells, not newly synthesized DNA [12]. Our results show that live cell numbers measured by the CCK-8 kit were increased by IGF-1 treatment. However, this method cannot differentiate whether the increase in live cells was due to new cell synthesis, reduced cell death, or both. In the current study, we applied a Click-iT EdU proliferation assay, which detects newly synthesized DNA. We found that increasing IGF-1 inhibited the proliferation of HTR8 cells in a dose-dependent manner. ID2 had the opposite effects of IGF-1 on trophoblast cell proliferation and differentiation, which confirms the fact that IGF-1 promotes HTR8 cell tube formation and migration through ID2 downregulation. This all supports the notion that ID2 is at least a partial mediator of IGF-1 function in trophoblast cells.

Besides ID2, there are many other factors that are involved in trophoblast cell proliferation and differentiation. In our study, ID2 overexpression only partially mitigated IGF-1’s effects, suggesting there could be an ID2-indpendent pathway which mediates the effects of IGF-1 on HTR8 cells. The complex cellular signaling pathways of IGF-1/IGF1-R include RAS/MEK/ERK and IRS-1/IRS-2/PI3K. We treated HTR8 cells with IGF-1 plus U0126 (an inhibitor of ERK1/2 phosphorylation [38]) or IGF-1 plus LY294002 (an inhibitor of PI3K [39]). However, blocking ERK or PI3K did not impact the effects of IGF-1 on ID2 expression, suggesting other pathways could be involved in IGF-1’s effect on ID2 in trophoblast cells.

## 5. Conclusions

Our study demonstrated that ID2 is likely a downstream mediator of IGF-1 in trophoblast cell proliferation and differentiation. Interventions in the IGF-1/ID2 signaling pathway may improve trophoblast cell biology and later-stage pregnancy outcomes.

## Figures and Tables

**Table 1:** Primers and probes for qRT-PCR.

Gene	Type	Sequence (5′–3′)
*h-ID2*		Hs04187239_m1 (Applied Biosystems)
*m-Id2*		Mm00711781-m1 (Applied Biosystems)
*h-GAPDH*	Forward	GAA GGT GAA GGT CGG AGT C
	Reverse	GAA GAT GGT GAT GGG ATT TC
	Probe	FAM-CA AGC TTC CCG TTC TCA GCC-TAMRA
*m-Actb*	Forward	AAG AGC TAT GAG CTG CCT GA
	Reverse	TGA TGG AAT TGA ATG TAG TTT CA
	Probe	TET-CAC TAT TGG CAA CGA GCG GTT CCG-TAMRA

## Data Availability

The original contributions presented in this study are included in the article/Supplementary Materials.

## Supplementary Materials

The following supporting information can be downloaded at: https://www.mdpi.com/article/10.3390/life14121663/s1, Figure S1: ID2 overexpressing plasmid elevates ID2 expression. Figure S2: Neither inhibition of MEK1/2 (U0126) nor inhibition of PI3K (LY-294002) abolishes the effects of IGF-1 on the expression of ID2.

## References

[1] Knöfler M., Haider S., Saleh L., Pollheimer J., Gamage T.K.J.B., James J. (2019). Human placenta and trophoblast development: Key molecular mechanisms and model systems. Cell. Mol. Life Sci..

[2] Surico D., Bordino V., Cantaluppi V., Mary D., Gentilli S., Oldani A., Farruggio S., Melluzza C., Raina G., Grossini E. (2019). Preeclampsia and intrauterine growth restriction: Role of human umbilical cord mesenchymal stem cells-trophoblast cross-talk. PLoS ONE.

[3] Selesniemi K., Albers R.E., Brown T.L. (2016). Id2 Mediates Differentiation of Labyrinthine Placental Progenitor Cell Line, SM10. Stem Cells Dev..

[4] Ling F., Kang B., Sun X.H. (2014). Id proteins: Small molecules, mighty regulators. Curr. Top. Dev. Biol..

[5] Florio M., Hernandez M.-C., Yang H., Shu H.-K., Cleveland J.L., Israel M.A. (2023). Id2 Promotes Apoptosis by a Novel Mechanism Independent of Dimerization to Basic Helix-Loop-Helix Factors. Mol. Cell. Biol..

[6] Gultice A.D., Selesniemi K.L., Brown T.L. (2006). Hypoxia Inhibits Differentiation of Lineage-Specific Rcho-1 Trophoblast Giant Cells. Biol. Reprod..

[7] Qi Z., Guo W., Zheng S., Fu C., Ma Y., Pan S., Liu Q., Yang X. (2019). Enhancement of neural stem cell survival, proliferation and differentiation by IGF-1 delivery in graphene oxide-incorporated PLGA electrospun nanofibrous mats. RSC Adv..

[8] Feng J., Meng Z. (2021). Insulin growth factor-1 promotes the proliferation and osteogenic differentiation of bone marrow mesenchymal stem cells through the Wnt/β-catenin pathway. Exp. Ther. Med..

[9] Liao S., Vickers M.H., Taylor R.S., Jones B., Fraser M., McCowan L.M., Baker P.N., Perry J.K. (2017). Maternal serum IGF-1, IGFBP-1 and 3, and placental growth hormone at 20 weeks’ gestation in pregnancies complicated by preeclampsia. Pregnancy Hypertens..

[10] Balachandiran M., Bobby Z., Dorairajan G., Gladwin V., Vinayagam V., Packirisamy R.M. (2021). Decreased maternal serum adiponectin and increased insulin-like growth factor-1 levels along with increased placental glucose transporter-1 expression in gestational diabetes mellitus: Possible role in fetal overgrowth. Placenta.

[11] Wilson R.L., Troja W., Sumser E.K., Maupin A., Lampe K., Jones H.N. (2021). Insulin-like growth factor 1 signaling in the placenta requires endothelial nitric oxide synthase to support trophoblast function and normal fetal growth. Am. J. Physiol. Regul. Integr. Comp. Physiol..

[12] Lai W., Yu L. (2023). Insulin-like growth factor 1 ameliorates pre-eclampsia by inhibiting zinc finger E-box binding homeobox 1 by up-regulation of microRNA-183. J. Cell. Mol. Med..

[13] Navarro M., Valentinis B., Belletti B., Romano G., Reiss K., Baserga R. (2001). Regulation of Id2 Gene Expression by the Type 1 IGF Receptor and the Insulin Receptor Substrate-1. Endocrinology.

[14] Graham C.H., Hawley T.S., Hawley R.C., MacDougall J.R., Kerbel R.S., Khoo N., Lala P.K. (1993). Establishment and Characterization of First Trimester Human Trophoblast Cells with Extended Lifespan. Exp. Cell Res..

[15] Cooney A.J., Takao T., Asanoma K., Kato K., Fukushima K., Tsunematsu R., Hirakawa T., Matsumura S., Seki H., Takeda S. (2011). Isolation and Characterization of Human Trophoblast Side-Population (SP) Cells in Primary Villous Cytotrophoblasts and HTR-8/SVneo Cell Line. PLoS ONE.

[16] Nandi P., Lim H., Torres-Garcia E.J., Lala P.K. (2018). Human trophoblast stem cell self-renewal and differentiation: Role of decorin. Sci. Rep..

[17] Li F., Kakoki M., Smid M., Boggess K., Wilder J., Hiller S., Bounajim C., Parnell S.E., Sulik K.K., Smithies O. (2018). Causative Effects of Genetically Determined High Maternal/Fetal Endothelin-1 on Preeclampsia-Like Conditions in Mice. Hypertension.

[18] Vatten L.J., Nilsen T.I.L., Juul A., Jeansson S., Jenum P.A., Eskild A. (2008). Changes in circulating level of IGF-I and IGF-binding protein-1 from the first to second trimester as predictors of preeclampsia. Eur. J. Endocrinol..

[19] Sun K., Visser A., Beijer M., Oudejans C.B.M., van Dijk M. (2018). The effect of maternal NODAL on STOX1 expression in extravillous trophoblasts is mediated by IGF1. PLoS ONE.

[20] Ayesha A., Bahnson E.M., Kayashima Y., Wilder J., Huynh P.K., Hiller S., Maeda-Smithies N., Li F. (2022). Vitamin B12 does not increase cell viability after hydrogen peroxide induced damage in mouse kidney proximal tubular cells and brain endothelial cells. Adv. Redox Res..

[21] Yang X., Ren L., Chen X., Pang Y., Jia B., Sun J., Quan X. (2023). BMP9 maintains the phenotype of HTR-8/Svneo trophoblast cells by activating the SDF1/CXCR4 pathway. BMC Mol. Cell Biol..

[22] Morales-Garza L.A., Puche J.E., Aguirre G.A., Muñoz Ú., García-Magariño M., De la Garza R.G., Castilla-Cortazar I. (2017). Experimental approach to IGF-1 therapy in CCl_4_-induced acute liver damage in healthy controls and mice with partial IGF-1 deficiency. J. Transl. Med..

[23] Schmittgen T.D., Livak K.J. (2008). Analyzing real-time PCR data by the comparative CT method. Nat. Protoc..

[24] Townley-Tilson W.H., Wu Y., Ferguson J.E., Patterson C. (2014). The ubiquitin ligase ASB4 promotes trophoblast differentiation through the degradation of ID2. PLoS ONE.

[25] Hwang S.-M., Sharma G., Verma R., Byun S., Rudra D., Im S.-H. (2018). Inflammation-induced Id2 promotes plasticity in regulatory T cells. Nat. Commun..

[26] Johnson A.L., Haugen M.J., Woods D.C. (2008). Role for inhibitor of differentiation/deoxyribonucleic acid-binding (Id) proteins in granulosa cell differentiation. Endocrinology.

[27] Jakubison B.L., Sarkar T., Gudmundsson K.O., Singh S., Sun L., Morris H.M., Klarmann K.D., Keller J.R. (2022). ID2 and HIF-1α collaborate to protect quiescent hematopoietic stem cells from activation, differentiation, and exhaustion. J. Clin. Investig..

[28] Liu F., Chen S., Yu Y., Huang C., Chen H., Wang L., Zhang W., Wu J., Ye Y. (2022). Inhibitor of DNA binding 2 knockdown inhibits the growth and liver metastasis of colorectal cancer. Gene.

[29] Zinina V.V., Ruehle F., Winkler P., Rebmann L., Lukas H., Möckel S., Diefenbach A., Mendez-Lago M., Soshnikova N. (2022). ID2 controls differentiation of enteroendocrine cells in mouse small intestine. Acta Physiol..

[30] Forbes K., Westwood M., Baker P.N., Aplin J.D. (2008). Insulin-like growth factor I and II regulate the life cycle of trophoblast in the developing human placenta. Am. J. Physiol. Cell Physiol..

[31] Frystyk J., Skjærbæk C., Dinesen B., Ørskov H. (2001). Free insulin-like growth factors (IGF-I and IGF-II) in human serum. FEBS Lett..

[32] Werner H., Bruchim I. (2009). The insulin-like growth factor-I receptor as an oncogene. Arch. Physiol. Biochem..

[33] Kadakia R., Arraztoa J.A., Bondy C., Zhou J. (2001). Granulosa cell proliferation is impaired in the Igf1 null ovary. Growth Horm. IGF Res..

[34] Ingec M., Gursoy H.G., Yildiz L., Kumtepe Y., Kadanali S. (2017). Serum levels of insulin, IGF-1, and IGFBP-1 in pre-eclampsia and eclampsia. Int. J. Gynecol. Obstet..

[35] Ning Y., Williams M.A., Vadachkoria S., Muy-Rivera M., Frederick I.O., Luthy D.A. (2004). Maternal plasma concentrations of insulinlike growth factor-1 and insulinlike growth factor-binding protein-1 in early pregnancy and subsequent risk of preeclampsia. Clin. Biochem..

[36] Lorenzini A., Salmon A.B., Lerner C., Torres C., Ikeno Y., Motch S., McCarter R., Sell C. (2013). Mice Producing Reduced Levels of Insulin-Like Growth Factor Type 1 Display an Increase in Maximum, but not Mean, Life Span. J. Gerontol. Ser. A Biol. Sci. Med. Sci..

[37] Irani M., Nasioudis D., Witkin S.S., Gunnala V., Spandorfer S.D. (2018). High serum IGF-1 levels are associated with pregnancy loss following frozen-thawed euploid embryo transfer cycles. J. Reprod. Immunol..

[38] Malik A., Pal R., Gupta S.K. (2017). Interdependence of JAK-STAT and MAPK signaling pathways during EGF-mediated HTR-8/SVneo cell invasion. PLoS ONE.

[39] Liu D., Luo D., Ge H., Zhang C., Wei S., Liang D., Tang D., Li J., Lin Y. (2022). Exposure to higher concentrations of exogenous ELABELA causes HTR-8/SVneo trophoblast cell dysfunction: A possible pathogenesis of pre-eclampsia. Pregnancy Hypertens..

