# Correction: Ssengonzi et al. Inhibitor of DNA Binding Protein 2 (ID2) Mediates the Anti-Proliferative and Pro-Differentiation Effects of Insulin-like Growth Factor-1 (IGF-1). *Life* 2024, *14*, 1663

**DOI:** 10.3390/life15071016

**Published:** 2025-06-26

**Authors:** Rebecca Ssengonzi, Yuye Wang, Jiayi Zhou, Yukako Kayashima, W. H. Davin Townley-Tilson, Balaji Rao, Qing Ma, Nobuyo Maeda-Smithies, Feng Li

**Affiliations:** 1 Department of Pathology and Laboratory Medicine, The University of North Carolina at Chapel Hill, Chapel Hill, NC 27599, USA; rssengo@email.unc.edu (R.S.); yuye@email.unc.edu (Y.W.); yukaya@email.unc.edu (Y.K.); davin_townley_tilson@med.unc.edu (W.H.D.T.-T.); maqing@email.unc.edu (Q.M.); nobuyo@med.unc.edu (N.M.-S.); 2 Department of Nutrition, Gillings School of Global Public Health, University of North Carolina at Chapel Hill, Chapel Hill, NC 27599, USA; jz0105@email.unc.edu; 3 Department of Chemical and Biomolecular Engineering, Golden LEAF Biomanufacturing Training and Education Center, North Carolina State University, Raleigh, NC 27606, USA; bmrao@ncsu.edu

In the original publication [1], there were mistakes in Tables 1 and 2. Methods, qRT-PCR. The correct version is as follows:

qRT-PCR: The total RNA from the cells was extracted using TRIzol Reagent (15596018, Invitrogen, Waltham, MA, USA) following the manufacturer’s instructions. A BioTek Synergy HT microplate reader was used to determine RNA concentration. mRNA was quantified with TaqMan real-time qRT-PCR (7500 real-time PCR system, Applied Bio-systems, Foster City, CA, USA) by using a one-step RT-PCR Kit (Bio Rad, Hercules, CA, USA) with GAPDH (human cells) and Actb (mouse tissues) as reference genes in each reaction. The 2^−ΔΔCt^ method was used for comparing the data [17,23]. Primer and probe sequences are listed in Table 1.

The authors state that the scientific conclusions are unaffected. This correction was approved by the Academic Editor. The original publication has also been updated.

## Figures and Tables

**Table 1:** Primers and probes for qRT-PCR.

Gene	Type	Sequence (5′–3′)
*h-ID2*		Hs04187239_m1 (Applied Biosystems)
*m-Id2*		Mm00711781-m1 (Applied Biosystems)
*h-GAPDH*	Forward	GAA GGT GAA GGT CGG AGT C
	Reverse	GAA GAT GGT GAT GGG ATT TC
	Probe	FAM-CA AGC TTC CCG TTC TCA GCC-TAMRA
*m-Actb*	Forward	AAG AGC TAT GAG CTG CCT GA
	Reverse	TGA TGG AAT TGA ATG TAG TTT CA
	Probe	TET-CAC TAT TGG CAA CGA GCG GTT CCG-TAMRA
