# Supplementary material for: Inhibitor of DNA Binding Protein 2 (ID2) Mediates the Anti-Proliferative and Pro-Differentiation Effects of Insulin-like Growth Factor-1 (IGF-1)
Source: Life (Basel). 2024 Dec 16;14(12):1663. doi: 10.3390/life14121663 (PMC11728018; doi:10.3390/life14121663)
Supplement: Supplementary file 1 [file life-14-01663-s001.zip › life-3355157-supplementary.pdf]

ONLINE SUPPLEMENT

Inhibitor of DNA Binding Protein 2 (ID2) Mediates the Anti-Proliferative and Pro-Differentiation Effects of Insulin-Like Growth Factor 1 (IGF-1)

Rebecca Ssengonzi[a], Yuye Wang[a], Jiayi Zhou[b], Yukako Kayashima[a], W.H. Davin Townley-Tilson[a], Balaji Rao[c], Qing Ma[a] ,Nobuyo Maeda-Smithies[a], Feng Li[a]*

[a] Department of Pathology and Laboratory Medicine, The University of North Carolina, Chapel Hill, NC 27599, USA.

[b] Department of Nutrition, Gillings School of Global Public Health, University of North Carolina at Chapel Hill, Chapel Hill, NC 27599, USA.

[c] Department of Chemical and Biomolecular Engineering, Golden LEAF Biomanufacturing Training and Education Center, North Carolina State University, Raleigh, North Carolina, USA.

*Correspondence to: Feng Li Ph.D. 703 Brinkhous-Bullitt Bldg, CB # 7525, University of North Carolina at Chapel Hill, NC 27599-7525

Phone: 919-597-0864

Fax: 919-966-8800

email: lif@med.unc.edu

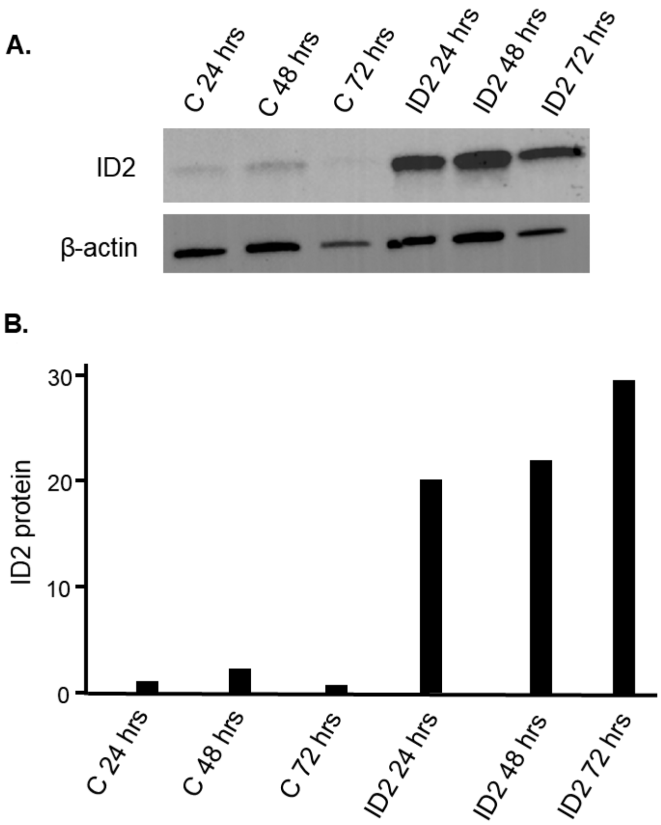

Supplementary Figure S1. ID2 overexpressing plasmid elevates ID2 expression. Time course experiments show ID2 protein (western blot in **A,** densitometric quantitation in **B**) are highest 72 hours after transfection which is when all functional assays were conducted.

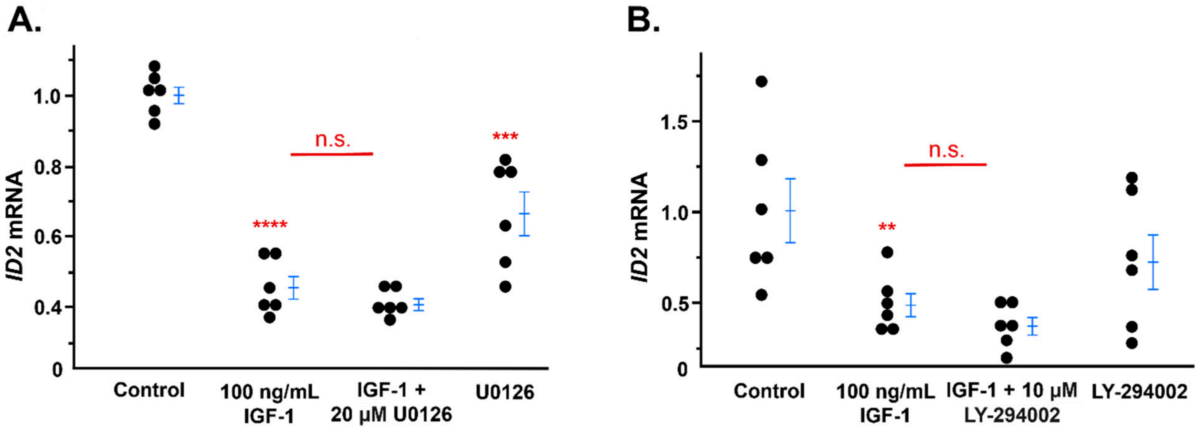

Supplementary Figure S2. Neither inhibition of MEK1/2 (U0126) nor inhibition of PI3K (LY-294002) abolishes the effects of IGF-1 on the expression of ID2. After 24 hours treatment with IGF-1 plus either U0126 (A) or LY-294002 (B) elevated mRNA levels of *ID2* in HTR8 cells. Experiments were repeated 2 times with each experimental group consisting of 3 replicates. ** $p < 0.01$, *** $p < 0.001$, **** $p < 0.0001$.
